# Parametric Analysis of Critical Buckling in Composite Laminate Structures under Mechanical and Thermal Loads: A Finite Element and Machine Learning Approach

**DOI:** 10.3390/ma17174367

**Published:** 2024-09-03

**Authors:** Omar Shabbir Ahmed, Jaffar Syed Mohamed Ali, Abdul Aabid, Meftah Hrairi, Norfazrina Mohd Yatim

**Affiliations:** 1 Department of Engineering Management, College of Engineering, Prince Sultan University, P.O. Box 66833, Riyadh 11586, Saudi Arabia; oahmed@psu.edu.sa; 2 Department of Mechanical and Aerospace Engineering, Faculty of Engineering, International Islamic University Malaysia, Kuala Lumpur 50728, Malaysia; meftah@iium.edu.my (M.H.); norfazrina@iium.edu.my (N.M.Y.)

**Keywords:** C-section channel, composite laminates, buckling analysis, FE analysis, machine learning

## Abstract

This research focuses on investigating the buckling strength of thin-walled composite structures featuring various shapes of holes, laminates, and composite materials. A parametric study is conducted to optimize and identify the most suitable combination of material and structural parameters, ensuring the resilience of structure under both mechanical and thermal loads. Initially, a numerical approach employing the finite element method is used to design the C-section thin-walled composite structure. Later, various structural and material parameters like spacing ratio, opening ratio, hole shape, fiber orientation, and laminate sequence are systematically varied. Subsequently, simulation data from numerous cases are utilized to identify the best parameter combination using machine learning algorithms. Various ML techniques such as linear regression, lasso regression, decision tree, random forest, and gradient boosting are employed to assess their accuracy in comparison with finite element results. As a result, the simulation model showcases the variation in critical buckling load when altering the structural and material properties. Additionally, the machine learning models successfully predict the optimal critical buckling load under mechanical and thermal loading conditions. In summary, this paper delves into the study of the stability of C-section thin-walled composite structures with holes under mechanical and thermal loading conditions using finite element analysis and machine learning studies.

## 1. Introduction

Due to their versatility and ease of integration with other materials, composites have become widely used in various applications [1]. In civil engineering, composites have demonstrated an interesting progression [2], finding application in material shaping and as a foundation for construction projects. Over time, civil engineers have become increasingly aware of the advantages offered by composite materials in construction. As a result, the use of composites in the building and construction industry has increased significantly. With growing demands for strength, safety, and reliability, composite engineering has become indispensable in numerous industries. Although composites may be more expensive than traditional construction materials, their benefits, such as being lightweight, corrosion-resistant, and stronger, outweigh the costs. The incorporation of fiber reinforcements in composites also provides excellent damping properties and remarkable fatigue resistance.

This knowledge enables engineers to utilize composite materials effectively in constructing reinforced structures capable of withstanding natural disasters like earthquakes and hurricanes. It is important to distinguish fiber-reinforced polymer (FRP) composites [3,4] from traditional construction materials like steel and aluminum because of their distinctive properties and applications. As we move forward, the role of composite engineering is predicted to expand further within civil engineering, playing a critical and increasingly important role in shaping the future of construction and infrastructure development.

## 2. Literature Review

The thin-walled member is considered one of the most efficient systems for preventing buckling due to its effective use of materials. By utilizing multiple thin-walled parts, this type of member allows for the creation of various shapes with a high shape factor, minimizing the amount of material required [2]. For example, the study conducted by Biagi et al. [5] has T-shaped thin-walled structures with varying geometries, which were examined for their use as ribs in aircraft. Also, Z-shaped composite thin-walled structures have been modeled through experiment work for the rib of aircraft [6]. One intriguing aspect of thin-walled structures is their capacity for self-improvement as well as the enhancement of other members. The decline in stiffness observed in laminated composites represents a noteworthy physical and mechanical reaction occurring in the context of damage and failure. To precisely investigate the mechanical characteristics of composite laminates [7], it becomes key to faithfully reproduce both the onset and the ensuing progression of this damage phenomenon [8].

To assess their influence on buckling, an investigation was conducted on thin-walled members featuring I-section cross-members with varying hole lengths and diameters. It was asserted that the length does not noticeably affect buckling [9], but the member’s load-bearing capacity is indeed influenced by hole dimensions. A member can sustain the critical load with hole diameter. Unlike prior research [10], this study does not specify a maximum hole width or number. Moreover, as explored in Guo [11], when investigating the correlation between slenderness ratio and stability, the presence of perforations in thin-walled members comes into focus. It is observed that as the size of the hole increases, the overall stability coefficient during buckling diminishes. However, for thin-walled members featuring multiple circular holes, the stability increases with greater spacing between the holes. Typically, in the face of compressive forces, individual plates within a thin-walled column buckle before the column experiences collapse [12]. It is common knowledge that a thin-walled structure is characterized by its thickness being considerably smaller than its height and width. Owing to its distinctive attributes, such as substantial strength and stiffness while maintaining low weight, this structural profile finds widespread utilization in contemporary industries, including aerospace, automotive, and construction sectors [13]. Buckling primarily results from lateral deflection in a structural member when subjected to axial forces, causing the column to deform due to its vulnerability. It is an abrupt and perilous failure. The column’s characteristics, such as length and strength, play a pivotal role in determining its susceptibility to buckling, especially in the case of long columns, which may undergo elastic buckling concerning their dimensions [14]. The classical Euler method employs linear analysis for column buckling calculations. For more accurate predictions of buckling loads, non-linear analysis offers a superior approach. This method utilizes non-linear static analysis to identify the point of instability as loads increase [15].

Numerous studies have been conducted on buckling in composites structures such as one on thermal buckling in laminates under uniform or non-uniform temperature fields, which was conducted [16] though FE simulations. Next, Shaterzadeh et al. [17] conducted research on the thermal buckling of composite plates with cutouts. However, the thermal buckling behavior of rectangular composite plates with square/rectangular and elliptical/circular cutouts has received less attention compared to other buckling modes. Such plates with cutouts typically experience membrane stresses from thermal or mechanical loads, making buckling a primary mode of failure [18]. An increase in temperature can lead to the buckling and reduced load-bearing capacity of a plate [19]. In their initial attempt to understand thermal buckling in plates, Gossard et al. [20] utilized the Rayleigh–Ritz method to analyze a simply supported rectangular plate made of homogeneous isotropic material, subjected to a tent-like temperature distribution. Investigating the buckling and post-buckling characteristics of thin plates, especially advanced composites with cutouts, is a fundamental area of research. Composite plates with cutouts are increasingly used in lightweight structures due to their impressive stiffness-to-weight and strength-to-weight ratios, offering significant weight reduction opportunities [21]. Cutout shapes in wing ribs can also be found effective in reducing the total weight under the structural and aerodynamics loads [22,23,24].

Distinct types of thin-walled structures find application across various industries. Previous studies have showcased the efficacy of integrating the extended FE formulation (XFEM) with genetic algorithms (GAs) for the efficient identification of structural defects. This approach, converging to actual defects, models the forward problem with XFEM and employs a GA as the optimization strategy. Chatzi et al. [25] have suggested enhancements to the XFEM-GA method, including a unique GA that expedites convergence and minimizes entrapment in local optima. Sliseris and Rocens [26] proposed a novel optimization method for composite plates with discretely variable stiffness. In a related perspective, innovative techniques to decrease the quantity of fitness function evaluations with GAs are presented by Park et al. [27] and are used in the transdisciplinary optimization. Furtado et al. [28] examined the innovative use of ML techniques in predicting statistical design allowances for composite laminates. In a separate initiative, Jung and Chang [29] developed a structural health monitoring (SHM) system for intelligent composite structures, integrating smart composite fabrics with embedded piezoelectric ribbon sensors for self-monitoring capabilities. Saberi et al. [30] employed deep learning to determine free vibration parameters of rectangular bistable composite plates, representing the initial application of deep learning to analyze such plate behavior. In the search of more efficient composite laminate design, Sorrentino et al. [31] introduced a theory-guided ML model that combines the Hashin failure theory with classical lamination theory.

This literature review underscores the active efforts to optimize laminated composites under various loading conditions. New techniques offer promising prospects for advanced analysis, especially when addressing different aspects of composite structures to achieve optimal designs. While existing optimization methods have primarily focused on GAs, the adoption of modern ML approaches for optimization remains limited. This highlights a significant opportunity for exploration and utilization to enhance the optimization process for laminated composites. Therefore, the current work focused on utilizing the different ML models for an optimal solution of selected parameters. Initially, the data were collected through the FE simulations by varying different parameters and their levels. A three-dimensional FE model was designed with and without holes and then the holes shapes were modified for analysis purposes under mechanical and thermal loads. Later, the analysis results were used for further investigation through the ML models.

## 3. Finite Element Method

The FE method is a numerical technique employed to solve partial differential equations (PDEs) governing the behavior of physical systems. In the FEM, a complex problem is divided into smaller, simpler regions or elements. By applying appropriate mathematical equations and principles, the behavior of each element and its interaction with neighboring elements can be modeled and analyzed.

### 3.1. Geometry and Modeling

**Figure 1:**
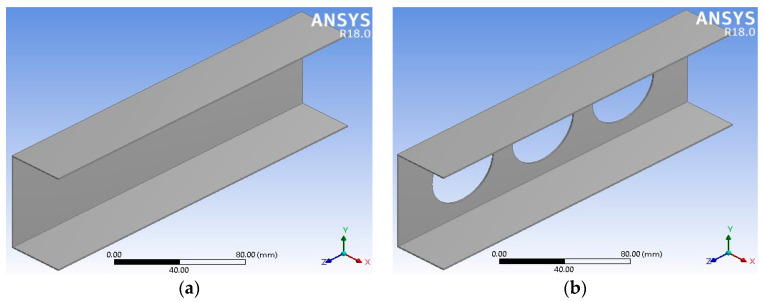
FE model (**a**) without holes and (**b**) with holes.

Using 3D ANSYS workbench software 2024 R1 [32], an FE model of the thin-walled structure is first created. Next, using meshing settings, the model is discretized into smaller parts. It is important to note that the mesh size and quality are critical parameters that can have a substantial impact on result accuracy [33]. In Figure 1, the model depicts a C-section channel crafted from glass fiber-reinforced polymer (GFRP), both with and without a hole. The channel’s flanges and web consist of eight layers of laminated sheets. Detailed dimensions of the C-section channel are presented in Table 1.

The FE model was used to investigate the effects of parameters and analysis on the laminated composite shell structure under mechanical and thermal loads. For this, ANSYS software 2024 R1 was used to simulate the results. The FE modeling for this research was conducted using the SHELL181 element. The SHELL181 element is a four-node element designed for analyzing shell structures ranging from thin to moderately thick. Each node of this element possesses six degrees of freedom, encompassing translation along the three *x*-, *y*-, and *z*-axes, including the rotation around the three *x*-, *y*-, and *z*-axes. The SHELL181 element offers support for full and reduced integration schemes within the element domain, considering the effects of distributed pressures, including follower (load stiffness). This element finds relevance in layered applications like composite shells and sandwich structures. The accuracy of modeling composite shells adheres to the first-order shear deformation theory. The formulation of this element relies on logarithmic strain and true stress metrics, permitting finite membrane strains like stretching while assuming minor curvature changes within each time increment.

**Figure 2:**
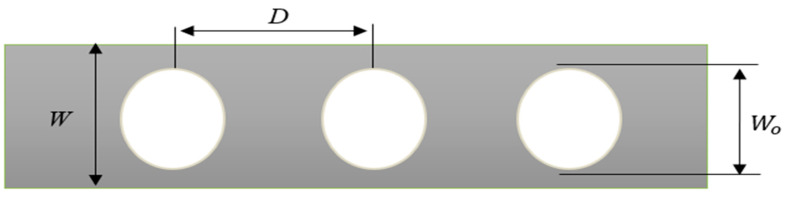
Description of opening and spacing ratio.

This study explored three parameters: spacing ratio, opening shape, and opening ratio, each examined across three distinct configurations. Figure 2 illustrates the circular hole and like this circular hole two other shapes of holes have been considered such as square and hexagonal. The spacing ratio and opening ratio are selected as follows:

(1) $$1.08<\frac{D}{W_{o}}<1.5$$

(2) $$1.25<\frac{W}{W_{o}}<1.75$$

where *D* is the distance between the two holes centroid; *W* is the total width of the plate (based on the Figure 2 front view, it is height); and *W*_0_ is the total width of the hole (based on the Figure 2 front view, it is height/diameter of the hole).

To establish the constitutive equations linking stresses and strains in the material, essential mechanical properties like Poisson’s ratio, shear modulus, and Young’s modulus were employed. Subsequently, the thermal properties of the composite structure, including the coefficient of thermal expansion, are defined. These properties play a key role in characterizing the mechanical and thermal responses under both mechanical and thermal loads. The laminate type of composites considered is quasi-isotropic, and the analysis involves layers of uniform thickness. This laminate type includes quasi-isotropic (0/90/45/−45)s, angle-ply (45/−45/45/−45)s, cross-ply (0/90/0/90/0/90/0/90), and balanced (−45/30/60/30/−30/45/−60/−30). The structures, along with their specific properties, were incorporated into the ANSYS workbench software. The analysis involved GFRP material with a mass density of 2200 kg/m^3^. To perform an analysis of composites, a sample rectangular plate has been modeled using the sample material which is illustrated in Table 2.

### 3.2. Boundary Conditions and Meshing

**Figure 3:**
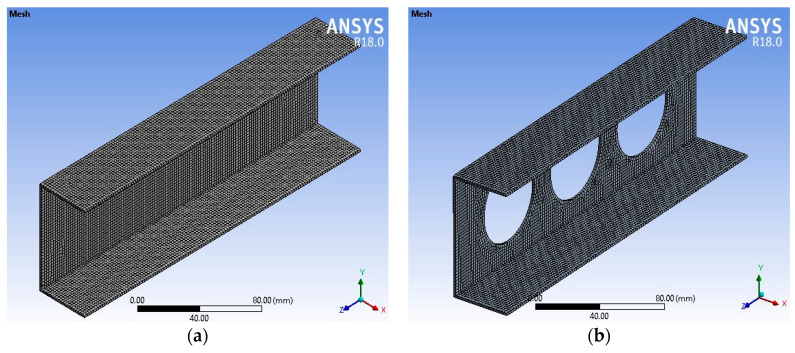
Models of the FE mesh (**a**) without holes and (**b**) with holes.

**Figure 4:**
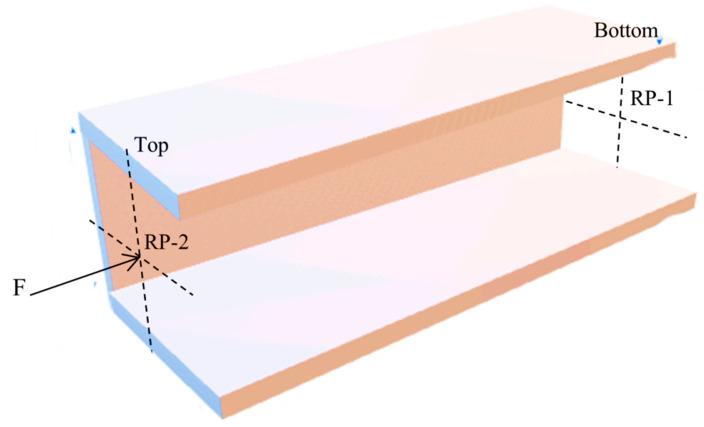
Loading conditions on the model.

To mesh the model (Figure 3), we simply chose a mesh model on the area and applied fine mesh with a minimum number of nodes and elements. Then, we refined the nodes to increase the fine mesh and mesh size. To mesh the model, a total of 81 elements (100 nodes) were formed for an element type 1 (SHELL181) which is chosen for the current FE model. The SHELL181 element has four nodes and the storage of layer data changes to all layers to increase the mesh. After conducting a mesh sensitivity analysis, a mesh size of 2 mm was chosen. The lower end of the column was fixed in all directions, and the load was applied to the upper part of the column at a designated reference point, as illustrated in Figure 4. RP-1 denoted the reference point at the bottom, while RP-2 was assigned to the upper section.

The buckling analysis utilized the linear perturbation procedure within the ANSYS workbench postprocessor for both mechanical and thermal loads. The models incorporated multi-layered QUAD4 and SHELL181 elements. For both load cases, the x, *y*, and *z* rotation degrees at the top end were fixed while permitting unrestricted movement along the vertical *z*-axis. Conversely, all degrees of freedom were restricted at the reference point at the bottom end to simulate fixed boundary conditions.

Rigid body-pin restrictions were applied to the upper nodes to apply a consistent load, approximating the contact of the strut with the top plate. For the mechanical load case, a nominal compressive load of 1.0 N was applied to the FE model [33,34]. Similarly, for the thermal load scenario, a uniform temperature of 150 degrees Celsius was applied across the entire structure, representing the change from room temperature (22 °C) to 150 °C.

To provide buckling mode shapes for both mechanical and thermal loads, the critical buckling load was determined by solving a linear eigenvalue buckling problem. Specifically, the first buckling mode—often referred to as the critical buckling load—was the focus of this process.

The buckling analysis utilized the linear perturbation procedure within the ANSYS workbench postprocessor. The models incorporated multi-layered QUAD4 and SHELL181 elements [34]. Fixing the *x*, *y*, and *z* rotation degrees at the top end while permitting unrestricted movement along the vertical *z*-axis was among the constraints imposed throughout the evaluation. On the other hand, all degrees of freedom were restricted at the reference point at the bottom end. Rigid body-pin restrictions were used to apply a consistent load to the upper nodes to approximate the contact of the strut with the top plate. The nominal compressive load applied to the FE model was 1.0 N. To provide buckling mode shapes for this investigation, the critical buckling load was determined by solving a linear eigenvalue buckling problem. Specifically, the first buckling mode—often referred to as the critical buckling load—was the focus of this process.

Finally, the total deformation (eigenvalue buckling) is obtained numerically, either directly or iteratively. The buckling load factor and mode shapes are determined as a solution. To extract the key buckling load factor and mode shapes, these findings are post-processed in ANSYS. The mode shapes help determine the kind of buckling that happened in the structure when it was subjected to being loaded either mechanically or thermally. In the meanwhile, a measure for estimating the safety factor and evaluating whether the structure is subject to buckling is the buckling load factor.

## 4. Machine Learning Approach

In this research, the FE model was validated through existing work and used to characterize the behavior of composite by various geometric and material parameters and a laminated interface. A formulated ML model was then developed for the composites. Design examples are provided to illustrate the effectiveness of the proposed ML model in describing the response to the release of the composite structures. A vulnerability analysis was then performed to examine the contribution of the selected predictors to the behavior of the composite. ML predictive models and reporting by the composite provide powerful tools for solving laminated structural problems in a variety of existing conventional composite structures.

**Figure 5:**
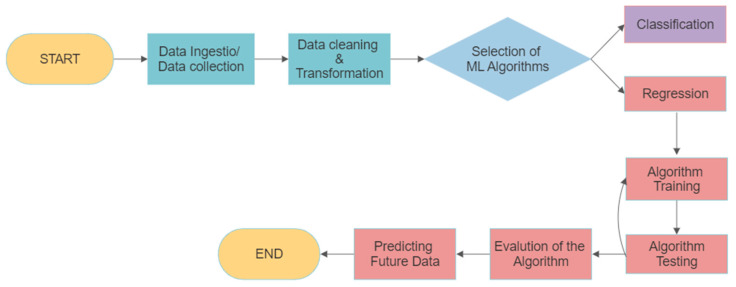
Process of machine learning in the current work.

In general, it is important to note with ML algorithms that they are random because of the arbitrary weight generated in the hidden layer. This can produce different accuracy depending on the random weights. Simple sequential heuristic search techniques are used to achieve the best accuracy. It is important to note that in ML the number of neurons in the hidden layer is randomly selected. In other words, there are no clear mathematical rules for deducing the right numbers of neurons to train. Huang et al. [35] have shown that with the differentiable activation functions, the number of neurons required in the hidden layer is smaller than the data size. In this application, some neurons select sigmoid as their activation function based on previous studies [36]. The overall used ML for the current work is illustrated in Figure 5.

The selection of regression models for predicting critical buckling loads in the dataset is aimed at exploring diverse modeling approaches that accommodate the mix of categorical features (such as orientation and shape) and continuous variables. Each model offers unique advantages from versatility in handling mixed data types, ability to capture complex relationships, high predictive performance, and the interpretability of linear regression to the non-linear pattern capturing ability of decision trees and ensemble methods like random forests and gradient boosting. This diverse set of models allows for a comprehensive analysis of the data, offering insights into both linear and non-linear relationships between the features and the target variable.

### 4.1. Regression Models

Regression analysis enables the quantification of this relationship between the outcome and associated variables. Various statistical prediction techniques have been developed, and for this study, four models were examined and compared: linear regression (LR), decision tree regression, AdaBoost decision tree regression, and gradient boosting regression (Table 3). When assessing the accuracy of predictions made by a model, it is essential to utilize evaluation metrics that can effectively measure the performance of a model in comparison to other models.

For evaluating regression performance, the commonly employed evaluation metric includes mean squared error, mean absolute error, explained variance score, and R-squared score. Regression models aim to create a mathematical equation, denoted as

(3) Y=f(X,w)

where Y represents the outcome (continuous or discrete) and X represents the independent variables. The unknown parameters w, also known as regression coefficients or weights, capture the relationship between the outcome and the variables. Moreover, in this research work different regression models have been selected to optimize the accuracy in a result. The error rates can be calculated using the equations of Table 4.

### 4.2. Design and Implementation

The dataset used in this analysis is obtained from the ANSYS workbench, where a C-section composite column is selected for this study. The purpose is to analyze the critical buckling load caused by both mechanical and thermal loads.

The data preparation process begins by loading the dataset containing various factors like opening ratio, shape, and the target buckling load using the pd.read_csv() function. We then split the data into a 70% training set and a 30% validation set to ensure our model generalizes well to unseen data. Examining the initial data structure is vital, so we use df.head() to gain a quick overview. Since some machine learning models require numerical representation for categorical features, we convert orientation and shape into numerical codes using as type (category) followed by cat.codes. Finally, we separate the features (opening ratio, spacing ratio, orientation, and shape) into the X variable and the target buckling load values into the Y variable for further analysis and modeling.

Data cleaning is an important step in data analysis, as missing, incomplete, or irrelevant data can introduce errors and bias into calculations and statistical functions. To ensure reliable results, we identify and address these issues. In this specific case, columns that do not significantly contribute to predicting the buckling load, such as the serial number column, are removed. It is worth noting that this dataset has no missing data, ensuring its completeness for analysis. By performing data cleaning and removing irrelevant columns, we streamline the dataset and focus on the relevant variables that influence the analysis, ultimately enhancing the accuracy and efficiency of subsequent data analysis tasks.

Data scaling plays a critical role in data preprocessing by aiming to normalize the features in a dataset and ensure they have a consistent scale and range. This process helps in improving the performance and stability of machine learning models. To perform data scaling, we utilize the StandardScaler class from the scikit-learn library. The process starts with creating an instance of the StandardScaler. Then, the scaler is fitted on the training data (X_train), which calculates the mean and standard deviation of each feature. Once fitted, the scaler can be used to transform both the training and validation data (X_train and X_test) by applying the computed mean and standard deviation. This transformation ensures that the features have a mean of 0 and a standard deviation of 1, resulting in a standardized scale across all features. By scaling the data, the model can effectively compare and interpret the features, as they are now on the same scale. This normalization step helps prevent certain features from dominating others due to their larger magnitudes, ultimately contributing to more accurate and reliable model training and predictions. In summary, data scaling using the StandardScaler enables the standardization of features by fitting the scaler on the training data and transforming both the training and test data using the computed mean and standard deviation. This process facilitates fair comparisons between features and promotes better performance of machine learning models.

While data scaling is valuable, it is not always necessary or universally beneficial. It highlights limitations like the outlier sensitivity of StandardScaler and explores alternative methods like MinMaxScaler and RobustScaler. In specific cases, scaling might not be needed. This understanding helps users make informed decisions based on their unique data and modeling goals.

## 5. Finite Element Results and Analysis

### 5.1. Mesh Independence Test

**Figure 6:**
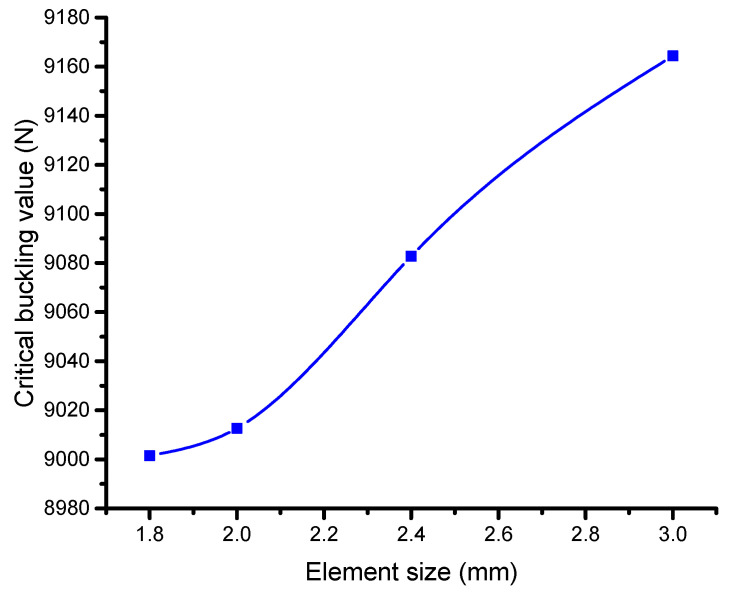
Mesh convergence study for C-section without cut-outs. Blue line is critical buckling load value and square point represents the exact value at a particular point.

A mesh convergence analysis, which focuses on a simple model without any cut-outs in the C-section channel, assesses the mesh correctness in the present model. Five element sizes, ranging in size from 1.8 mm to 3 mm, are evaluated to investigate the effects of various mesh sizes. For more examination, the element size that produces the most comparable outcomes is selected. Then, in the present work, mesh refinement is evaluated with respect to a particular example, like a previous study. According to the investigation, the element size of 2 mm exhibits the lowest error and leads to result convergence, closely matching the reference value. Consequently, all models employ this 2 mm element size, with minor tweaks made to provide smoother borders around the holes in the structure. As indicated in Figure 6, the 2 mm element size produces the lowest error, while maintaining reasonable computational time with this mesh configuration. These findings suggest that further refinement is unnecessary, and errors are minimized [34].

### 5.2. Validation of Simulation Model

To validate the current simulation model of Doan and Thai [34], work has been repeated through the ANSYS software and the determined value of the critical buckling load. To ensure accuracy in the current work, the length of the specimen and cross-sectional dimensions were exactly duplicated to match those in their study. To evaluate the dependability of the model, the buckling behavior of the quasi-isotropic laminate and of the angle-ply laminate FE models was compared to the findings of corresponding numerical analyses. For the quasi-isotropic laminate, [0/+45/+45/+45/+45]s were the stacking sequences used. To assure more dependability, the hole-free beam model was first compared for both laminates. Table 5 displays the critical buckling load values along with a comparison with the prior findings of this study. Based on the current simulation, it has been found that the ANSYS simulation has less error (2.56%) with experimental work as compared to the previous ABAQUS 2021 simulation (8.6%), and this is because of high accuracy in mesh selection, which has been found through the mesh independence test.

The current model under thermal load is further validated through a comparison with theoretical and numerical results [38]. The University of Connecticut’s theoretical solution for isotropic materials is contrasted with the FE results for the first mode. A previous study examining an FRP composite thin rectangular plate with in-plane dimensions of 2 m length and 1 m breadth served as the foundation for the construction of the FE model. For a given scenario, the thickness of the plate is determined by the length-to-thickness ratio (s = a/t) using symmetric quasi-isotropic stacking sequences (90/45/−45/0/0/−45/45/90). The comparison between the FE results and the theoretical solution is presented in Table 6.

### 5.3. Total Deformation

**Figure 7:**
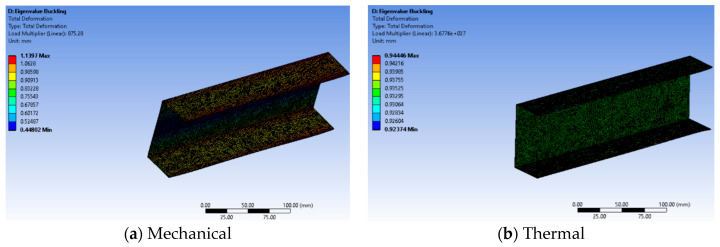
Total deformation (eigenvalue buckling) in the absence of cutouts.

**Figure 8:**
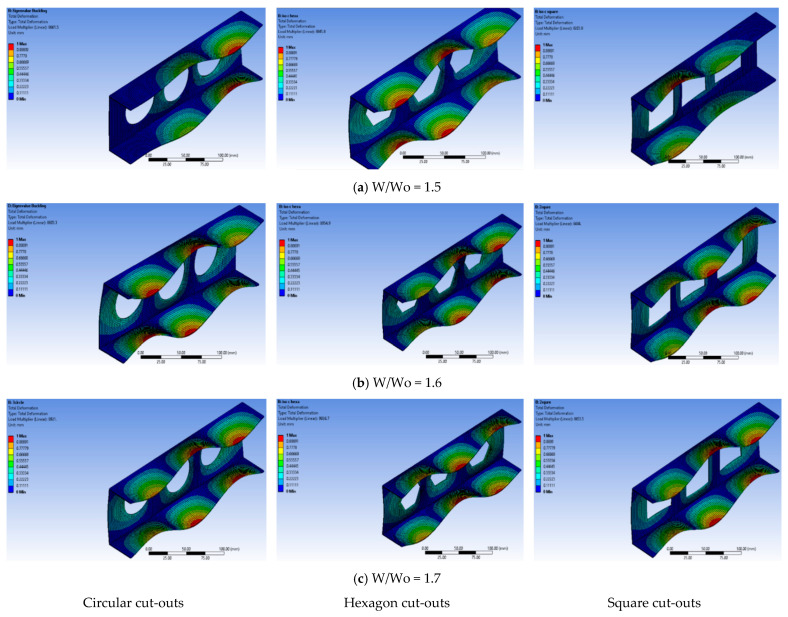
Total deformation (eigenvalue buckling) for circular cut-outs under mechanical load.

**Figure 9:**
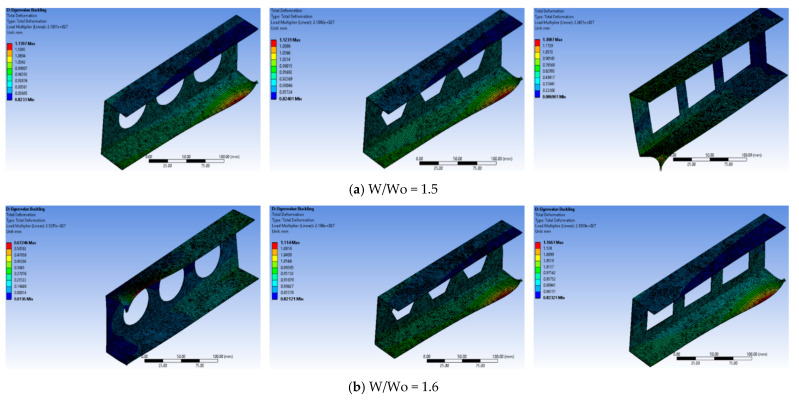

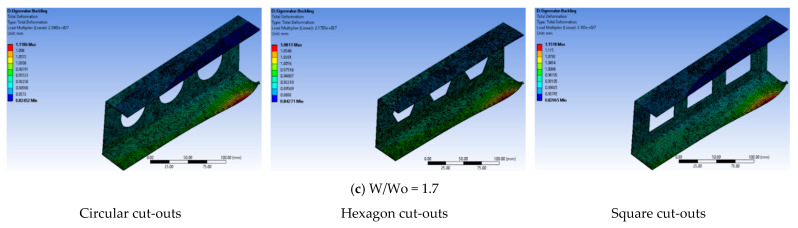
Total deformation (eigenvalue buckling) for circular cut-outs under thermal load.

The simulation results from ANSYS illustrate a notable reduction in the load-carrying capacity of a beam in the presence of apertures, ultimately leading to failure. Eigenvalue data depicting the critical buckling load and critical thermal buckling for various models, with and without apertures, are presented in Figure 7, Figure 8 and Figure 9. Specifically, the eigenvalues of the C-section channel without apertures are shown in Figure 7, indicating that these values are expected to be greater than those of the C-section channel with apertures. This observation implies that the C-section channel without apertures can endure a higher load before experiencing buckling.

To enhance the strength of the beam while reducing its weight, the channel incorporates apertures and is subjected to testing with various shapes and sizes (Figure 7). These tests help to determine the effects of different aperture configurations on the structural integrity and load-bearing capacity of the beam. Overall, the findings from the ANSYS simulation underscore the importance of carefully designing aperture placements and shapes to maintain the structural integrity of beams and prevent premature failure under axial loads. This research aims to guide the development of more robust and efficient beam designs for various engineering applications.

The significance of the contour results lies in their ability to reveal the distinct behavior of the beam under mechanical and thermal loads, allowing for a comprehensive understanding of its structural response. By extracting contours independently for both conditions, engineers can observe significant variations in eigenvalues, which are critical in determining the buckling load capacity. Despite the similarity in deformation patterns between the mechanical and thermal load cases, the presence of apertures in the C-sectional channel results in a noticeable decrease in buckling load capacity. This reduction is clear in the plotted data, indicating that apertures have a substantial impact on the ability of the beam to withstand external forces before buckling occurs. Understanding these contour results is vital for design optimization, as engineers can make informed decisions about aperture configurations to enhance structural integrity and load-carrying capacity. It helps identify regions of stress concentration and potential failure points, guiding the development of safer and more efficient beam designs. Additionally, this knowledge aids in choosing suitable materials and reinforcement strategies to counteract the negative effects of apertures, ensuring the overall reliability and longevity of the beam in practical applications.

This study investigated three different cases with W/Wo ratios of 1.5, 1.6, and 1.7 to understand the effects of cut-out configurations on the buckling behavior of C-sectional channels. Among these cases, the ratio of 1.3 stood out due to its larger cut-out diameter, which resulted in a lower buckling load and reduced structural stability. However, it is essential to consider that this scenario also had a lower channel weight, indicating a cut-off between weight and buckling load capacity. Conversely, cases with smaller cut-out diameters exhibited increased buckling loads, implying that channels with higher weights can sustain greater loads without buckling when cut-outs are absent. Furthermore, this study compared three different cut-out shapes, with the circular cut-out demonstrating superior strength compared to that of the other two shapes.

The circular cut-out has an advantage that arises from its lack of sharp edges, enabling a more even distribution of stress and enhancing overall structural integrity. In contrast, the square cut-out, characterized by 90-degree angles, is more susceptible to immediate structural failure due to stress concentration at these sharp corners. These findings are vital for structural design optimization, as they highlight the impact of cut-out diameter and shape on buckling behavior and channel weight. Engineers can use this knowledge to select appropriate configurations based on specific application requirements. By considering the cut-offs between weight, buckling load, and structural stability, they can make informed decisions to create safer and more efficient C-sectional channel designs. Moreover, understanding the influence of cut-out shape helps prevent the presence of stress concentration points that could compromise the structural integrity of the beam, ensuring long-term reliability and safety in real-world engineering applications.

The obtained results from this study reveal interesting findings regarding the buckling behavior of thin-wall structures with and without perforations. In all the models analyzed, the flanges displayed a half-wave deformation along the longitudinal axes, attributed to the compressive stresses experienced during the loading process. This phenomenon was consistent across the reference model (without any holes) and the perforated models. The reference model, lacking any perforations, exhibited clear local buckling along the web. On the other hand, in the perforated models, buckling was not only observed along the web but also near the edges of the holes. This suggests that the presence of perforations influences the buckling behavior, leading to localized buckling phenomena near the hole boundaries. Interestingly, despite the introduction of perforations and variations in their shapes for quasi-isotropic materials, a consistent pattern of half-wave deformation was observed in all models. This implies that the laminate configuration plays a significant role in determining the critical buckling load and the buckling shape of the thin-wall structure. Different laminate configurations can result in diverse buckling behaviors, influencing the structural stability of the thin-wall structure. Furthermore, it was noted that the first mode of buckling observed in all models was Euler buckling, affecting both the web and flanges of the thin-wall structure. Euler buckling refers to the sudden buckling failure of slender columns under axial compression, and its presence in all models highlights its dominance in the structural response of the thin-wall beams.

This study demonstrates that the buckling behavior of thin-wall structures is influenced by the presence of perforations and the laminate configuration. The half-wave deformation pattern along the flanges and the occurrence of localized buckling near hole edges in perforated models suggest that aperture placement can significantly affect structural stability. Moreover, the consistent occurrence of Euler buckling across all models underscores its importance in predicting buckling failures in thin-wall structures. These findings have implications for design optimization and provide valuable insights for engineering applications where thin-wall structures with apertures are commonly used.

### 5.4. Effect of Parameters

In this section, the effect of each parameter is determined through the graphical representation for the case of mechanical and thermal load. This study does not show the comparison of results with respect to loading conditions. Since both loads have different responses such as mechanical load, the critical buckling load can be calculated from the compressive force in newton (N). Meanwhile, the thermal critical buckling load is computed via the effect of compressive force when the temperature effect deforms the C-section channel (T-critical).

#### 5.4.1. Quasi-Isotropic

**Figure 10:**
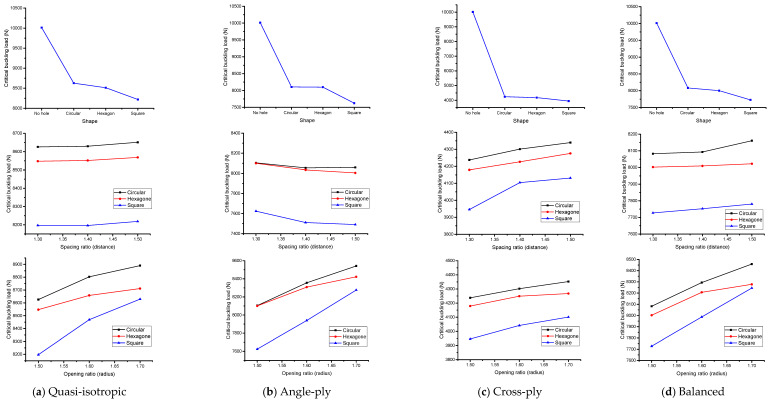
Influence of each parameter under mechanical load.

**Figure 11:**
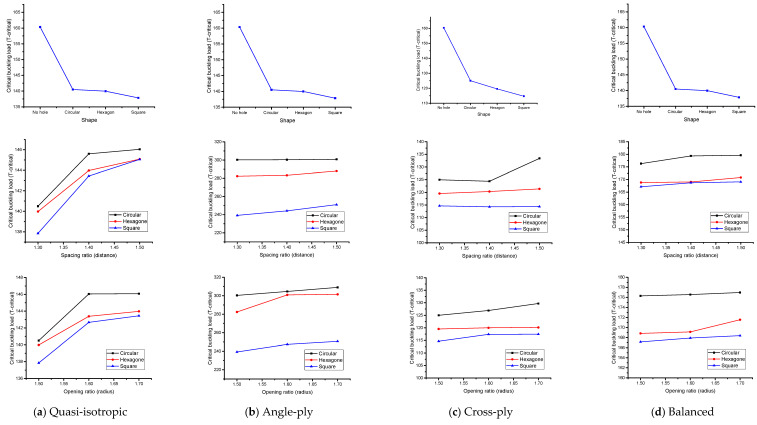
Influence of each parameter under thermal load.

Figure 10a and Figure 11a exhibit the impact of various cut-out forms, spacing, and opening ratios under both mechanical and thermal stresses. The influence of cut-outs while keeping constant spacing and opening ratios is the major focus of the inquiry. The inclusion of cut-outs results in a reduction in the buckling load, with the circular cut-out presenting the greatest critical buckling load and the square cut-out having the lowest capacity. Notably, the addition of holes results in a more noticeable decrease in load, which is explained by a larger area removal on the web of the thin wall. The square cut-out shows the lowest buckling load under mechanical or thermal stresses, whereas the circular form has a larger critical buckling load capacity since it involves the least amount of area reduction. Furthermore, Figure 10a and Figure 11a depict the relationship between the opening ratio and buckling load. A decrease in the opening ratio implies larger holes in the thin-wall GFRP composite member, aligning with the trend observed earlier. Larger holes negatively impact the buckling load capacity, as they remove more material from the web, weakening the structure’s ability to withstand mechanical or thermal loads. The findings underscore the significance of considering opening size in assessing the structural behavior of thin-wall composite members. Mechanical loads show the higher variation in buckling load in every example analyzed in this work, whereas thermal loads show less variation. On the other hand, the critical buckling load stays comparatively constant, as shown in Figure 10a and Figure 11a. When the structure has a large percentage of solid sections and the diameter is in the low range, a spacing ratio of 1.5 exhibits greater buckling values under thermal stress circumstances. In the end, Figure 10a and Figure 11a demonstrate a negligible decline in trend with a decreasing opening ratio. The distance between the centers of two holes decreases while the opening ratio is constant, suggesting that a smaller ratio results in a lower buckling strain. For all laminates, the largest critical buckling load is found when a circular shape, 1.7 opening ratio, and 1.5 spacing ratio are combined. This is true for both mechanical and thermal stresses. The combined impact of these characteristics will be further explored in the sections that follow.

#### 5.4.2. Angle-Ply

This study as shown in Figure 10a and Figure 11a is replicated in Figure 10b and Figure 11b, which show the combined effect of various laminates and cut-out forms. When cut-outs are incorporated, the buckling load decreases noticeably. The square cut-out has the lowest load capacity in terms of buckling, while the circular cut-out consistently shows the greatest critical buckling load. The addition of holes causes a particularly noticeable decrease in load capacity, mostly because the web of the thin wall loses more surface. The circular shape minimizes this removal of area, resulting in a higher critical buckling load. Notably, the square cut-out shape consistently exhibits the lowest buckling load under mechanical or thermal loads. Furthermore, Figure 10b and Figure 11b reveal a similar trend in the angle-ply model as observed in the quasi-isotropic model results. A declining trend is evident as the opening ratio decreases, emphasizing the inverse relationship between larger holes and smaller ratios. This aligns with the earlier observation that increased perforation on the thin wall’s web leads to a decrease in the buckling load. Mechanical loads consistently provide the greatest values of buckling loads across all scenarios studied, whereas thermal loads show the lowest range. The critical buckling load is shown to be essentially consistent throughout a range of spacing ratios in Figure 10b and Figure 11b. A spacing ratio of 1.5 indicates higher buckling values under thermal stress circumstances, as shown in Figure 11b. This is especially true when the diameter is in the low range and the structure’s body has a large percentage of solid sections. Moreover, Figure 10b and Figure 11b illustrate a minor decline as the opening ratio decreases. This suggests that a decrease in the ratio leads to a reduced distance between the centers of two holes. After a thorough analysis of every figure covered in this part, it can be said that the configuration including a circular shape, 1.7 opening ratio, and 1.5 spacing ratio consistently shows the greatest critical buckling stress, regardless of the laminate utilized. This is true for loads that are mechanical as well as thermal. Furthermore, it is observed that a larger distance inside the structure correlates to a higher buckling load, while lowering the distance leads to a drop in buckling stress. The impact of these elements taken together will be further examined in the sections that follow.

#### 5.4.3. Cross-Ply

The results presented in Figure 10c and Figure 11c depict the behavior of a cross-ply fiber orientation in terms of shape, opening, and spacing ratio under mechanical and thermal load. Overall, the trends observed in this model are like those in the previously discussed models, with variations in the numerical values of the critical buckling load. The maximum critical buckling load reaches approximately 10,000 N when there is no hole in the channel, while the structures that have a circular hole have values that suddenly decrease to 4200 N which shows a good variation compared to that of the previous two models. On the other hand, the lowest point is found to be around 3900 N, as shown in Figure 10c. Similarly, the results for thermal buckling load in this model resemble the findings from the previous models, as indicated in Figure 11c. The impact of the spacing ratio in the cross-model reveals similarities among all the models examined in this study. When considering the square shape, the minimum critical buckling load value of approximately 3950 N is observed with a spacing ratio of 1.3. However, with a spacing ratio of 1.5, the critical buckling load increases to around 4050 N in Figure 10c. This indicates that an increase in the spacing ratio leads to an increment in the critical buckling load under both mechanical and thermal loads, which is illustrated in Figure 10c and Figure 11c.

#### 5.4.4. Balanced

The results of the balanced model in this study exhibit similarities with the outcomes of other models such as the quasi-isotropic, angle-ply, and cross-ply models. The effects of different parameters, including shape, spacing ratio, and opening ratio, were investigated in Figure 10a–c and Figure 11a–c, respectively, and the behavior of the balanced model was consistent with the trends observed in previous models. In terms of the effect of shape, the critical buckling load under both mechanical and thermal loads was found to be like that of previous studies. This indicates that the shape of the apertures has a limited impact on the buckling behavior, and the overall trends observed in the balanced model align with those of previous models in Figure 10d and Figure 11d. For the effect of spacing ratio, the critical buckling load in the balanced model was notably higher than in the previous case, exceeding 8000 N (Figure 10d). This indicates that the load-carrying capacity of the balanced model is significantly stronger, making it capable of efficiently supporting thin-walled structures. Under thermal load conditions (Figure 11d), the T-critical values were in the range of 160 to 175, which is like the observations for the spacing ratio. Finally, the results of the opening ratio in the balanced model also demonstrated higher critical buckling loads under both mechanical and thermal loads. This further supports the idea that the balanced model, along with its specific orientations, exhibits superior strength-to-weight ratios, making it a valuable option for designing structures in engineering applications. Overall, the balanced model shows promising characteristics, offering high strength with low weight. Its critical buckling loads are comparable to or even exceed those of other established models. This suggests that the balanced model can serve as a reliable and efficient option for designing thin-walled structures, especially when considering the performance under both mechanical and thermal loading conditions. Engineers and designers can leverage these findings to optimize thin-walled structures with apertures, enhancing their load-carrying capacity and structural integrity for various engineering applications.

## 6. Machine Learning Model Evaluation and Results

In the model training and evaluation phase, all regression models are applied to analyze a dataset. The process involves fitting the model using scaled training features (X_train_scaled) and corresponding labels (y_train) with the sklearn.linear_model modules for all regression class. Predictions are made on the test set using the predict() method. The model performance is assessed through metrics such as mean squared error (MSE), root mean squared error (RMSE), and R-squared (R^2^).

**Figure 12:**
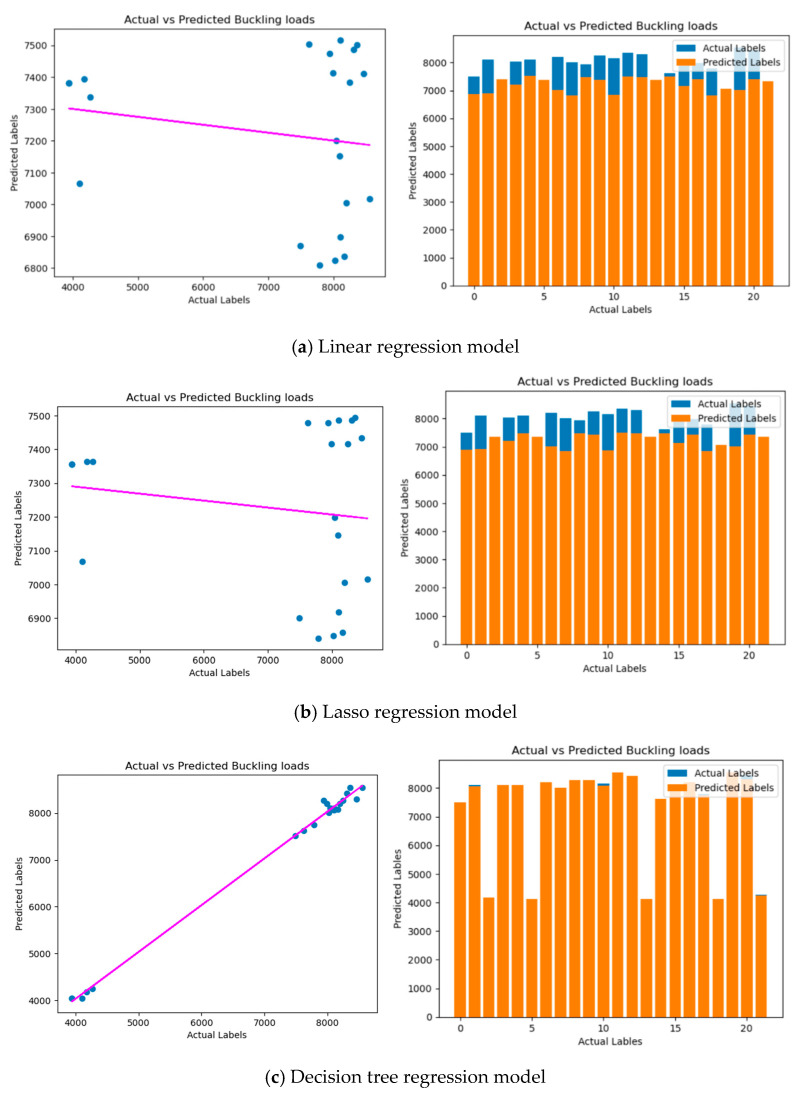

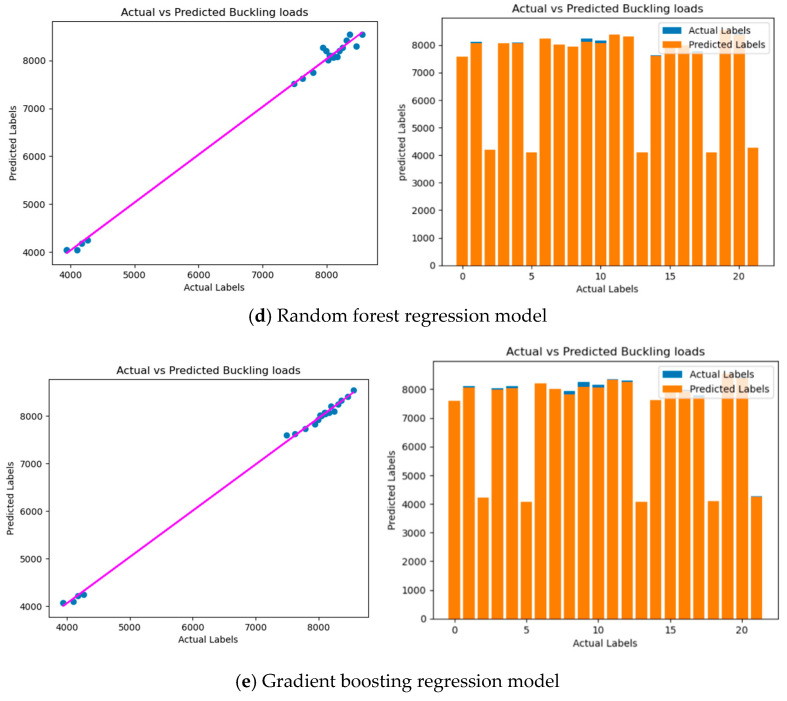
Analysis of regression model under mechanical load. Pink—predicted labels and blue—actual label.

**Figure 13:**
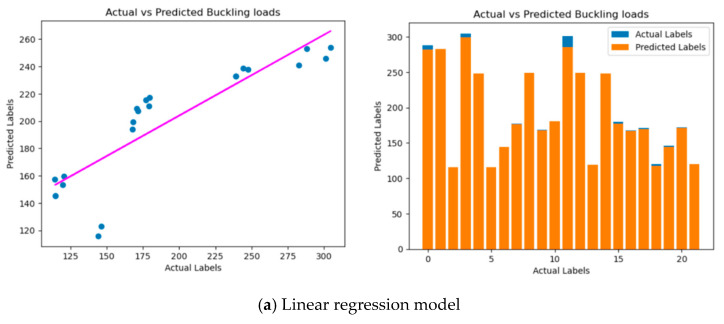

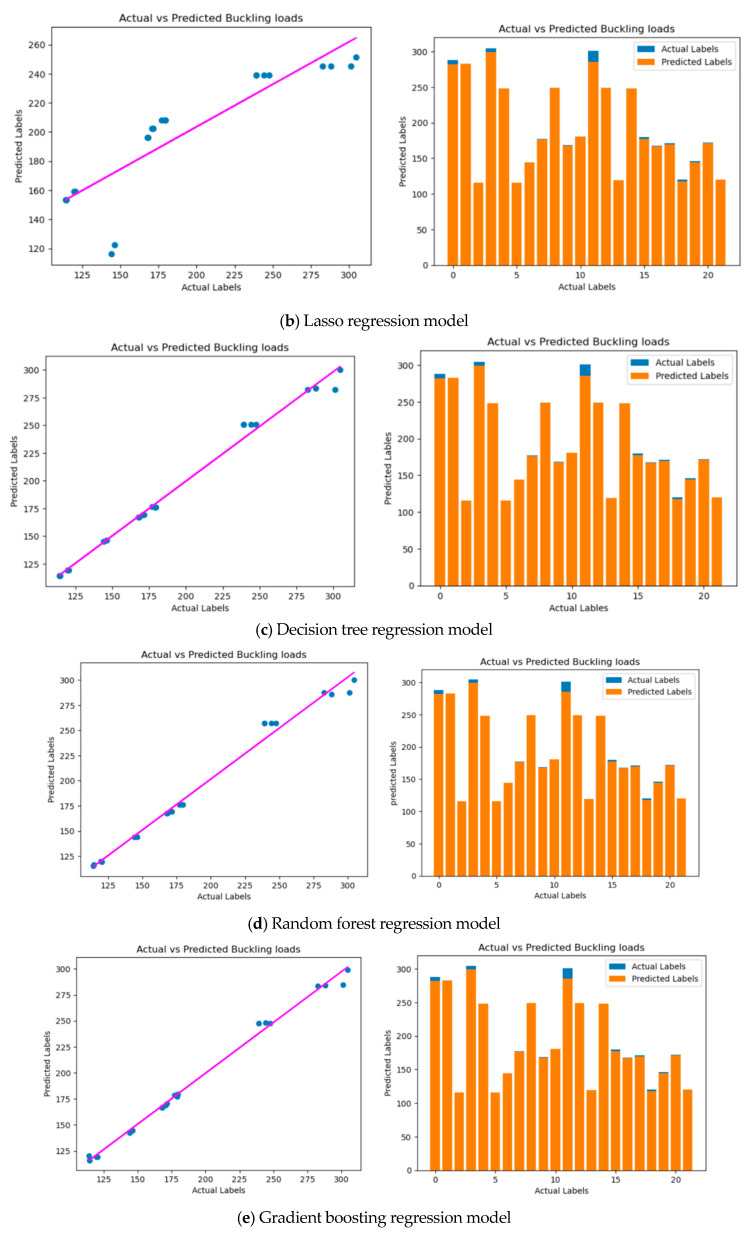
Analysis of regression model thermal load. Pink—predicted labels and blue—actual label.

For the regression model (Figure 12 and Figure 13), MSE gauges the squared variance between predicted and actual values, with lower values indicating greater precision. R^2^ quantifies the proportion of predictable variance in the dependent variable, where values closer to 1 suggest a better model fit. The calculated MSE, RMSE, and R^2^ are displayed, and a scatter plot is created to visualize the predictions of the model versus actual values. This comprehensive analysis aids engineers and researchers in evaluating the reliability of a model for predicting critical buckling loads based on the dataset, enabling informed decision-making and potential model improvements for more accurate engineering analyses.

Across various regression models used for predicting critical buckling loads, several key performance metrics are employed to assess their reliability and precision. These metrics are critical for engineers and researchers, as they provide valuable insights into the model’s performance, aiding in informed decision-making and potential model improvements for more accurate engineering analyses. In the assessment of all regression models under both loading conditions, including linear regression, lasso regression, decision tree regression, random forest regression, and gradient boosting regression, the MSE serves as a critical measure to gauge the squared variance between predicted and actual values. Lower MSE values indicate greater precision, highlighting the model’s ability to accurately predict critical buckling loads. Additionally, the RMSE provides an error measurement in the same units as the target variable, enhancing interpretability. Engineers and researchers can use the RMSE to assess the models’ performance in a way that directly relates to the practical context of their work. The R^2^ value is another common metric used to quantify the proportion of predictable variance in the dependent variable. Values closer to 1 suggest a superior model fit, indicating that the model can explain a substantial portion of the variance in critical buckling loads.

To provide a clear visual comparison of predicted labels against actual ones, scatter plots are created for all regression models. These plots offer insights into the models’ predictive performance, with a well-performing model demonstrating data points clustered around a diagonal line, signifying a strong correlation. This comprehensive analysis empowers engineers and researchers to evaluate the reliability and suitability of each regression model for predicting critical buckling loads in various scenarios. It also opens the door to potential enhancements for more accurate and efficient engineering analyses, regardless of the specific regression approach employed.

The analysis employs the MSE, RMSE, and R-squared to assess the performance of buckling load prediction models. A lower MSE and RMSE indicate better accuracy, signifying the model’s ability to minimize the difference between predicted and actual loads. The RMSE, being in the same units as the target variable, offers easier interpretation for engineers. R-squared reflects the model’s ability to explain the variance in critical buckling loads, with values closer to 1 suggesting a better fit. These metrics guide model selection based on specific goals: prioritizing accuracy favors models with a lower MSE and RMSE, while interpretability might favor models less sensitive to outliers, potentially favoring a high mean absolute error (MAE). However, R-squared should not be the sole deciding factor as it does not account for model complexity or overfitting. Understanding these nuances helps justify the chosen metrics and their necessary role in selecting the most suitable model for predicting critical buckling loads.

Through the assessment of various ML algorithms, encompassing linear regression, lasso regression, decision tree regression, and random forest regression, their respective performances in predicting the critical buckling load of thin-walled structures were evaluated. This evaluation relied on essential metrics, including the MSE, RMSE, and R^2^ values.

The analysis employs metrics like the MSE, RMSE, and R-squared to assess model performance, but a deeper understanding within the context of predicting critical buckling loads is critical. Firstly, connecting these metrics to engineering significance is vital. A lower MSE and RMSE directly translate to reduced risk of structural failure by minimizing the difference between predicted and actual buckling loads. This emphasizes the practical importance of achieving better accuracy in ensuring the safety and integrity of structures during engineering design. Secondly, acknowledging the limitations of R-squared is important. While it provides insights into model fit, high values might not guarantee reliable predictions for extreme load scenarios, which are often critical in engineering. Finally, considering the potential influence of outliers is essential. If the dataset is susceptible to them, their impact on the chosen metrics and the interpretation of model performance should be discussed. Exploring robust alternatives like the MAE might be necessary for a more reliable assessment, especially if outliers are a significant concern.

Additionally, visual comparisons were facilitated through scatter plots. In comparison to FE analysis, it was discerned that all ML algorithms yielded reasonably precise predictions of the critical buckling load. The models consistently exhibited low MSE and RMSE values, underscoring the proximity of predictions to the actual critical buckling loads. Additionally, high R^2^ values were obtained, suggesting that a significant proportion of the variance in the critical buckling loads could be explained by the models. These results are promising as they align well with the outcomes of the FE analysis, indicating that the chosen ML algorithms can effectively capture the complex behavior of thin-walled structures under different loading conditions. The advantage of using ML algorithms, such as random forest regression and gradient boosting regression, lies in their ability to handle non-linear relationships and capture intricate interactions among variables. As thin-walled structures often exhibit complex behavior due to geometric configurations and material properties, these algorithms can offer more accurate predictions than traditional linear regression approaches.

The application of ML algorithms to predict critical buckling loads in thin-walled structures can significantly benefit engineering applications. Firstly, these algorithms can save computational time and resources compared to FE analysis, as they provide rapid predictions once trained. FE analysis involves solving complex differential equations, which can be time-consuming and computationally expensive for large-scale models. ML algorithms, once trained on suitable datasets, can quickly predict critical buckling loads for various design configurations, allowing engineers to explore a wide range of possibilities efficiently. Moreover, ML algorithms can help in the optimization of thin-walled structures. Engineers can use these algorithms to identify the key design parameters and their effects on the critical buckling load. By conducting sensitivity analyses, they can determine the most influential factors and optimize the structural design to enhance the load-carrying capacity while minimizing weight. Furthermore, ML algorithms can assist in the early-stage design process, where only limited data or preliminary designs are available. Engineers can use existing data and ML models to make informed decisions and guide the initial design iterations. As more data become available from physical testing or simulations, the ML models can be continuously refined and improved. The successful application of various ML algorithms in predicting the critical buckling load of thin-walled structures demonstrates their effectiveness and potential in engineering applications. By providing accurate and efficient predictions, ML algorithms can significantly aid in the design optimization and decision-making process, leading to safer, more reliable, and cost-effective thin-walled structures in various engineering fields.

Selecting appropriate ML models for predicting critical buckling loads requires careful consideration of various factors. This analysis demonstrates the suitability of linear regression, decision tree regression, random forest regression, and gradient boosting regression, each offering unique strengths.

- Linear regression, as a fundamental and interpretable model, serves well as a baseline and is suitable for linear or linearized relationships. Its strength lies in providing clear interpretations of coefficients, aiding in understanding feature impact.
- Decision trees excel at capturing nonlinear relationships and interactions in the data, making them suitable for complex scenarios. They are robust to outliers and handle diverse data types without extensive preprocessing.
- Random forests, by combining multiple decision trees, effectively address overfitting and generalize well, especially in high-dimensional or noisy data. They offer robustness to outliers and missing values while requiring minimal hyperparameter tuning.
- Gradient boosting, through sequential model building, excels at capturing complex patterns and achieving high predictive accuracy. It effectively handles various data types and is suitable for a wide range of regression problems.

These models offer a balance between interpretability, flexibility, and predictive performance, allowing for comprehensive exploration of critical buckling load relationships and providing valuable insights for engineering decision-making.

### 6.1. Hyperparameter Tuning (Grid Search)

In the hyperparameter tuning stage, a grid search is conducted on the gradient boosting regression model using the GridSearchCV class. Here is how it works: First, a parameter grid is defined to specify various combinations of hyperparameters, including the learning rate and the number of estimators. Then, an instance of the GridSearchCV class is instantiated from the ‘sklearn.model_selection’ module, with the gradient boosting regression model and the parameter grid passed as arguments. Grid search is initiated by invoking the ‘fit()’ method on the GridSearchCV object, with the training data (‘X_train’ and ‘y_train’) provided. This exhaustive search explores each combination within the hyperparameter grid, evaluating them based on the specified scoring metric to select the best one. The optimal values for the learning rate and number of estimators are obtained from the ‘best_params_’ attribute of the GridSearchCV object. Subsequently, the model is retrained using the best hyperparameters identified from the grid search to ensure optimal settings. Predictions are then made on the test set (‘X_test’) using the ‘predict()’ method of the retrained model. These evaluated metrics offer insights into the accuracy and goodness of fit of the tuned model. Additionally, a scatter plot is generated to visually compare predicted labels against actual labels, facilitating a graphical assessment of the model’s performance. Through these steps, GridSearchCV facilitates the systematic exploration of different hyperparameter combinations for the gradient boosting regression model, leading to improved predictions for the critical buckling load based on the provided dataset.

The hyperparameters tuned in the gradient boosting regression model using grid search were the learning rate and the number of estimators (n_estimators). These hyperparameters were selected to optimize the model’s performance in predicting the critical buckling load of thin-walled structures under mechanical load. The GridSearchCV class from scikit-learn was employed to perform an exhaustive search over a predefined parameter grid, evaluating each combination and selecting the best one based on the specified scoring metric (in this case, the R^2^ score).

The parameter grid specified the following hyperparameters and their corresponding values to be explored:

1. Learning rate: [0.1, 0.5, 1.0];
2. Number of estimators (n_estimators): [50, 100, 150].

The grid search iteratively tested all possible combinations of these hyperparameter values, evaluating the performance of a model using cross-validation. The best combination of hyperparameters was determined based on the R^2^ score. The best parameter combination identified by the grid search was a learning rate of 0.1 and 50 estimators (n_estimators). This process allowed for the systematic exploration of different hyperparameter combinations, ultimately leading to the selection of the optimal settings for the gradient boosting regression model to achieve improved predictions of critical buckling loads based on the given dataset.

### 6.2. Machine Learning Result Matrix

The results obtained from each step of the methodology may vary depending on the dataset and specific hyperparameters used. Detailed results for each model can be provided based on the specific dataset and implementation. Meanwhile, the MSE, RMSE, and R^2^ are commonly used performance metrics in regression tasks [39]. Here is an explanation of each metric: R^2^ ranges from 0 to 1, where 0 indicates that the model does not explain any variation in the target variable, and 1 indicates a perfect fit. A higher R^2^ value suggests better model performance, but it should be interpreted in the context of the specific problem and data. Meanwhile, the MSE and RMSE measure the magnitude of the prediction errors, and R^2^ provides an indication of the proportion of variance explained by the model. These metrics should be considered together to assess the overall performance and fit of the regression model.

Table 7 presents the evaluation results of different ML algorithms for predicting the critical buckling load of thin-walled structures under mechanical load. The following metrics were calculated for each model: the MSE, RMSE, and R^2^ value. Both linear regression and lasso regression models have relatively high MSE and RMSE values and negative R^2^ values. This suggests that these models do not perform well in predicting the critical buckling load, as the predicted values are far from the actual values. The negative R^2^ values indicate that these models do not explain much of the variance in the critical buckling load. The decision tree regression model shows significantly lower MSE and RMSE values compared to those of the linear-based models, indicating better accuracy in predicting the critical buckling load. The high R^2^ value of 0.996 suggests that this model explains a substantial portion of the variance in the critical buckling load. The random forest regression model performs slightly worse than the decision tree model, with slightly higher MSE and RMSE values. Nevertheless, it still demonstrates good accuracy with an R^2^ value of 0.9951. The gradient boosting regression model outperforms all other models in terms of accuracy. It exhibits the lowest MSE and RMSE values and the highest R^2^ value of 0.9978, indicating an excellent fit to the data and accurate predictions of the critical buckling load. The gradient boosting regression model is the most accurate among the evaluated models, providing the most reliable predictions for the critical buckling load of thin-walled structures. This model’s performance is significantly better than that of the linear-based models (linear regression and lasso regression) and comparable to that of the decision tree and random forest models, but with even higher accuracy and goodness of fit. The gradient boosting regression model is, therefore, the preferred choice for predicting the critical buckling load in engineering applications, as it offers high accuracy and efficiency in handling complex relationships between variables.

Table 8 presents the evaluation results of different ML algorithms for predicting the critical buckling load of thin-walled structures under thermal load. The following metrics were calculated for each model: the MSE, RMSE, and R^2^ value. Both linear regression and lasso regression models have moderate MSE and RMSE values, indicating a reasonable level of accuracy in predicting the critical buckling load under thermal load. The R^2^ values of 0.7163 and 0.7226 suggest that these models explain a significant proportion of the variance in the critical buckling load under thermal conditions. The decision tree regression model outperforms the linear-based models, with significantly lower MSE and RMSE values. The high R^2^ value of 0.9911 indicates that this model explains most of the variance in the critical buckling load under thermal load, making it an excellent fit to the data. The random forest regression model performs slightly worse than the decision tree model but still exhibits good accuracy. The MSE and RMSE values are higher than those of the decision tree model, but the R^2^ value of 0.9854 shows that this model still explains a substantial portion of the variance in the critical buckling load under thermal conditions. The gradient boosting regression model is the most accurate among all the evaluated models. It shows the lowest MSE and RMSE values and the highest R^2^ value of 0.9940, indicating a superior fit to the data and highly accurate predictions of the critical buckling load under thermal load.

The gradient boosting regression model is the most accurate and reliable among the evaluated models for predicting the critical buckling load under thermal load conditions. It outperforms the linear-based models (linear regression and lasso regression) and offers slightly better accuracy than the decision tree and random forest models. The gradient boosting regression model is, therefore, the preferred choice for predicting the critical buckling load in engineering applications under thermal load, as it provides highly accurate and efficient predictions in handling the complexities of thermal loading conditions. While this study explores the potential of machine learning for predicting critical buckling loads, it primarily focuses on presenting the foundational aspects of this application.

The application of machine learning algorithms for predicting critical buckling loads in thin-walled structures presents both practical implications and challenges in real-world engineering applications. Firstly, machine learning enables improved design optimization by efficiently navigating the complex design space of thin-walled structures, thereby enhancing both their efficiency and safety. Moreover, by leveraging machine learning, engineers can potentially save significant time and costs by reducing the need for costly physical testing, thus accelerating the design process. This not only expedites product development but also allows for more iterations and the exploration of design alternatives. Additionally, accurate predictions facilitated by machine learning algorithms are pivotal in ensuring the structural integrity of thin-walled structures, consequently enhancing safety and regulatory compliance. However, these advancements come with their set of challenges. One such challenge is the interpretability of machine learning models, as understanding the factors influencing predictions is critical for engineers to make informed decisions during the design process. Furthermore, the transferability and generalization of these models across different scenarios are paramount for their widespread adoption in engineering practice. Addressing these implications and challenges will be instrumental in enabling engineers to effectively harness the power of machine learning to optimize thin-walled structure designs, thereby ensuring safety, efficiency, and regulatory compliance in real-world applications.

## 7. Conclusions

This research conducted a comprehensive analysis of the buckling behavior of thin-walled C-section channels under mechanical and thermal loads, considering the presence of holes. Finite element method modelling was employed to simulate critical buckling load factors and mode shapes for various scenarios. The findings indicated that the presence of holes significantly influenced the structural behavior, with distinct mode shapes and lower critical buckling load factors in cases with holes, highlighting the importance of considering holes in the design of C-section channels. Parametric studies further explored the impact of factors like hole shapes, sizes, positions, and material composition on the structural behavior under different loads, providing valuable insights for engineering decisions and optimization. The research introduced machine learning models for predicting critical buckling loads, achieving high accuracy across various models, with gradient boosting followed by decision tree and random forest models standing out. These models offer practical applications for structural engineers in assessing C-section column stability under different loads and input parameters, aiding in real-world design and optimization. In summary, this study contributes to the understanding of buckling behavior in thin-walled structures and emphasizes the importance of accounting for holes in C-section channel design. The combination of FE modelling, parametric studies, and machine learning provides valuable tools for improving structural integrity and performance in practical engineering applications. Further research can explore additional hole types and their effects on thin-walled structure buckling behavior.

## Figures and Tables

**Table 1:** C-section channel dimensions.

Description	Size
Thickness (flange and web) per layer	0.125 mm
Total thickness	1 mm
Height	80 mm
Width	40 mm
Length	250 mm
No. of layers	8
Orientation	4 types

**Table 2:** Material properties of the GFRP material.

Parameter	GFRP Material [34]
Density	2200 kg/m^3^
Poisson’s ratio (ʋ_12_ = ʋ_13_ = ʋ_23_)	0.29
Young’s modulus (E_1_)	58 GPa
Young’s modulus (E_2_) = E_3_)	10 GPa
Shear modulus (G_12_)	8 GPa
Shear modulus (G_13_) = (G_23_)	5 GPa
Material type	Sample material

**Table 3:** Regression models.

Serial No.	Models	Key Equation
4	Linear regression	y=β0+β1x1+β2x2+⋯+βnxn
5	Lasso regression	y=β0+β1x1+β2x2+⋯+βnxn+λ∑|βi|
6	Decision tree regression	y=f(x)
7	Random forest regression	y=f₁(x)+f₂(x)+⋯+fₙ(x)
8	Gradient boosting regression	y=f₁(x)+f₂(x)+⋯+fₙ(x)

**Table 4:** Error rates.

Serial No.	Error Types	Key Equation
1	Mean square error (MSE)	$MS(y,\;\overset{-}{y})=(1/n)\;*\;\sum \;(yi-\overset{-}{y}i)²$
2	Root mean square error (RMSE)	$RMSE(y,\;\overset{-}{y})=\surd ((1/n)\;*\;\sum \;(yi-\overset{-}{y}i)²)$
3	R-square	$R²(y,\;\overset{-}{y})=1-[(1/n)\;*\;\sum \;(yi-\overset{-}{y}i)²]\;/\;[(1/n)\;*\;\sum \;(yi-\overset{-}{y})²]$

**Table 5:** Validation of FE model under mechanical load.

Laminate Types	Structural Type	Critical Buckling Load (N)			Error (%)
		Experimental [34]	ABAQUS [37]	Present Work ANSYS	
Quasi-isotropic	Without hole	10,500 N	11,201 N	10,237 N	2.56 and 8.6
Quasi-isotropic	With hole	-	8551 N	8625 N	0.86

**Table 6:** Validation of FE model under thermal load.

Configuration of Model (Thickness Ratio t/b)	Critical Buckling Temperature		Error (%)
	Theoretical [38]	Present Work Numerical	
Quasi-isotropic	28 °C	26 °C	7.14

**Table 7:** Evaluation matrix for mechanical load.

Sl. No.	Model	MSE	RMSE	R^2^
01	Linear regression	3,330,969.06	1825.09	−0.1615
02	Lasso regression	3,295,965.72	1815.48	−0.1501
03	Decision tree	11,093.85	105.33	0.9960
04	Random forest	13,016.04	114.09	0.9951
05	Gradient boosting	6115.63	78.20	0.9978

**Table 8:** Evaluation matrix for thermal load.

Sl. No.	Model	MSE	RMSE	R^2^
01	Linear regression	1127.64	33.5804	0.7163
02	Lasso regression	1104.25	33.2303	0.7226
03	Decision tree	35.0631	5.9214	0.9911
04	Random forest	57.7908	7.6020	0.9854
05	Gradient boosting	23.7657	4.8750	0.9940

## Data Availability

The data presented in this study are available on request from the corresponding author (the data are not publicly available due to privacy or ethical restrictions).
